# Resveratrol analogue 4,4′-dihydroxy-*trans*-stilbene potently inhibits cancer invasion and metastasis

**DOI:** 10.1038/srep19973

**Published:** 2016-02-01

**Authors:** Monica Savio, Daniela Ferraro, Cristina Maccario, Rita Vaccarone, Lasse D. Jensen, Federica Corana, Barbara Mannucci, Livia Bianchi, Yihai Cao, Lucia Anna Stivala

**Affiliations:** 1 Department of Molecular Medicine, Immunology and General Pathology Unit, University of Pavia, Pavia, Italy; 2 Department of Microbiology, Tumour and Cell Biology, Karolinska Institute, Stockholm, Sweden; 3 Department of Biology and Biotechnology, Comparative Anatomy and Citology Laboratory, University of Pavia, Pavia, Italy; 4 Department of Medical and Health Sciences, Linköping University, Linköping, Sweden; 5 Centro Grandi Strumenti, University of Pavia, Pavia, Italy

## Abstract

We investigated the preventive effects of resveratrol analogue 4,4′-dihydroxy-*trans*-stilbene (DHS) on cancer invasion and metastasis. Two different *in vivo* approaches of mouse and zebrafish lung cancer invasion models were employed in our study. The *in vitro* results showed that DHS displays potent inhibition on anchorage-dependent or -independent cell growth of LLC cells, leading to impairment of the cell cycle progression with reduction of cell numbers arresting at the G1 phase, an evident accumulation of pre-G1 events correlated with apoptotic behaviour. In addition, DHS induces a marked inhibition of LLC cell migration and matrigel invasion. In a murine lung cancer model, tumour volume, cell proliferation, and tumour angiogenesis were significantly inhibited by DHS. Importantly, liver metastatic lesions were significantly reduced in DHS-treated mice. Similarly, DHS significantly inhibits lung cancer cell dissemination, invasion and metastasis in a zebrafish tumour model. These findings demonstrate that DHS could potentially be developed as a novel therapeutic agent for treatment of cancer and metastasis.

During the past two decades, resveratrol (3,5,4′-trihydroxy-*trans*-stilbene, RSV) has received considerable attention due to its wide spectrum of biological functions, and especially its cancer chemoprevention activity, that has been extensively reviewed^1^,^2^,^3^,^4^,^5^. A large number of structure-activity studies have revealed molecular determinants of RSV, which are required for its specific effects^6^,^7^,^8^. Consequently, its chemical scaffold has been used as starting point to synthesize new molecules with enhanced cytotoxic, antiproliferative and anti-tumour properties^9^,^10^,^11^,^12^. Our first investigations on RSV analogues demonstrated the importance of introducing into the molecular skeleton a hydroxyl group in 4′ position of the aromatic ring, together with the *trans*-conformation, in inducing antiproliferative response^6^. The synthesis of the RSV analogue 4,4′-dihydroxy-*trans*-stilbene (DHS) allowed us to confirm the higher efficiency of this analogue, both as antioxidant and antiproliferative agent^9^,^13^. DHS was for the first time found as a metabolite of the *trans*-stilbene excreted in the urine of guinea pigs, rabbits and mice^14^,^15^,^16^ and, some years later, isolated from the MeOH extract of bark of *Y. periculosa*^17^. Subsequently, the estrogenic activity of *trans*-stilbene was attributed to the formation of hydroxylated metabolites, among which DHS^16^. Almost at the same time, DHS was synthesized and identified as specific estrogen receptor (ER)-ligand^18^, and later, as an ERα antagonist by inducing slow and selective proteasome-independent down-regulation of the receptor in two estrogen-sensitive breast cancer cell lines^19^. Interestingly, both 4- and 4′-OH have been demonstrated to be critical for the ability of DHS to induce ER down-regulation^19^. Similarly, *orto*- or *para*-hydroxyl functionalities have been demonstrated to possess high antioxidative activity^20^,^21^,^22^. In fact, DHS presents, among the different RSV analogues, the strongest antioxidant capacity toward lipoperoxyl radicals^23^, and higher than that resveratrol against free radical-induced peroxidation of both rat liver microsome^22^ or human erythrocyte ghosts^24^. More recently, a compound developed by elongating of the resveratrol conjugated links, and bearing 4,4′-dihydroxy groups, displays a strong increase in antioxidant, cytotoxic and apoptosis-inducing activities compared to the parental molecule^25^. Furthermore, DHS has been demonstrated to inhibit normal and cancer cell proliferation^9^,^13^, matrix metalloproteinase-2 and -9 production/activity^13^,^26^, endothelial cell migration and angiogenesis^26^ with higher efficiency than resveratrol. We have recently demonstrated that DHS markedly inhibits breast cancer cell adhesion to the extracellular matrix components as well as their migration and invasion, probably dependent on E-cadherin modulation^13^; once again, DHS but not RSV, markedly suppressed chemical-induced cell transformation of BALB/c 3T3 mouse fibroblasts, confirming a potential role of the two 4, 4′ hydroxyl groups of the stilbenic backbone in preventing *in vitro* cell transformation^13^.

The *in vivo* anticancer effects exerted by RSV have been widely reviewed^2^,^3^,^27^, while comparatively fewer *in vivo* studies have investigated those RSV derivatives possessing, in *in vitro* systems, enhanced anti-tumour activity^28^,^29^. No evidence is available, till date, on DHS and its antitumour capacity studied through *in vivo* models. Using C57BL/6J mouse bearing a tumour resulting from an implantation of primary Lewis Lung Carcinoma (LLC) cells, we show that the resveratrol analogue DHS reduces the size of the primary tumour, the angiogenesis process and the number of liver metastasis. Similarly, in the zebrafish metastasis model *Tg(fli1:*EGFP), DHS reduces markedly dissemination, invasion and metastasis of LLC cells, indicating that this resveratrol-analogue holds enormous potential as an anti-tumour compound.

## Results

### DHS induces lung cancer cell death

**Figure 1:**
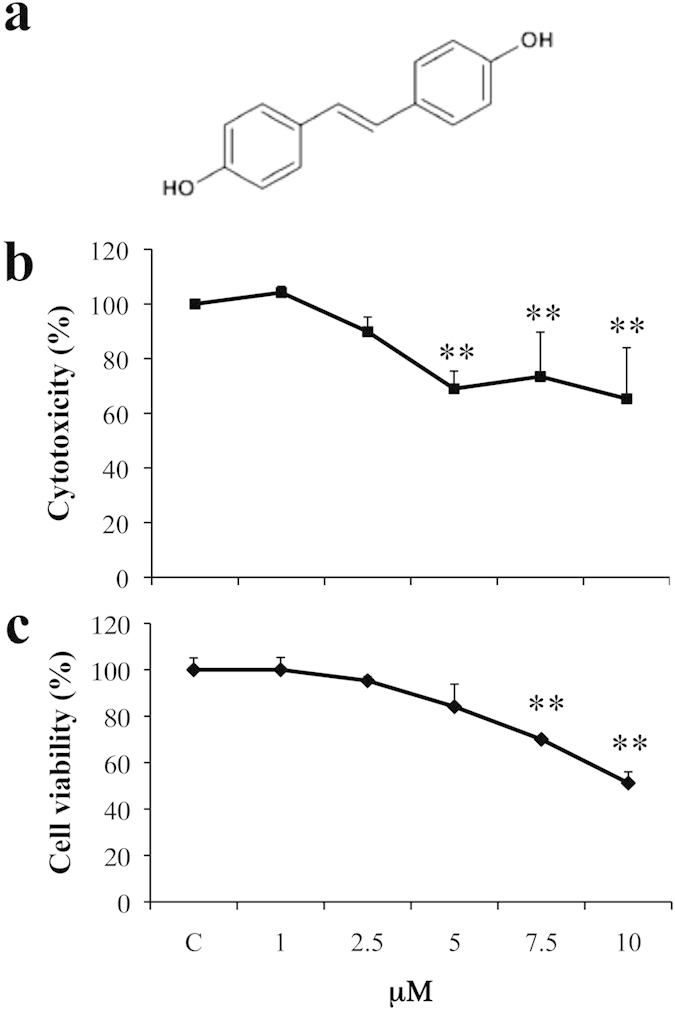
Cytotoxicity of DHS. (**a**) Chemical structure of DHS. (**b**) The cytotoxicity was determined by the MTT assay after 24 h of treatment with the indicated concentrations of DHS. The reduction of MTT by active mitochondria, which results in a colour change measured at 570 nm with a microplate reader, was used as an indicator of viable cell number. (**c**) Cell viability was determined by the Trypan Blue staining. Unstained (viable) and stained (non-viable) cells were counted in a Burker chamber, and the results were expressed as a percentage of viable versus the total number of counted cells. Data are the means ± SD from at least three independent experiments: values are expressed as percentages and are relative to untreated control cells (**p ≤ 0.01 compared with control cells).

DHS is characterized by the presence of two hydroxyl groups in the *para*-positions 4 and 4′ (Fig. 1a). Its effect on LLC cell viability was determined by using MTT-based colorimetric assay after 24 h incubation period with increasing concentrations (range 1–10 μM) (Fig. 1b). At the lower concentrations of 1 and 2.5 μM, DHS didn’t induce any significant effect on LLC cells, while an initial cell death rate of about 32% was found at the concentration as high as 5 μM. This percentage remained almost similar at both 7.5 and 10 μM (Fig. 1b). Trypan blue staining confirmed that the observed cytotoxic effect was related to cell death. In fact, a dose-dependent reduction in cell viability of about 30% and 49% was detected at 7.5 and 10 μM, respectively (Fig. 1c).

### DHS impairs LLC cell proliferation, cell cycle distribution and induces apoptosis

**Figure 2:**
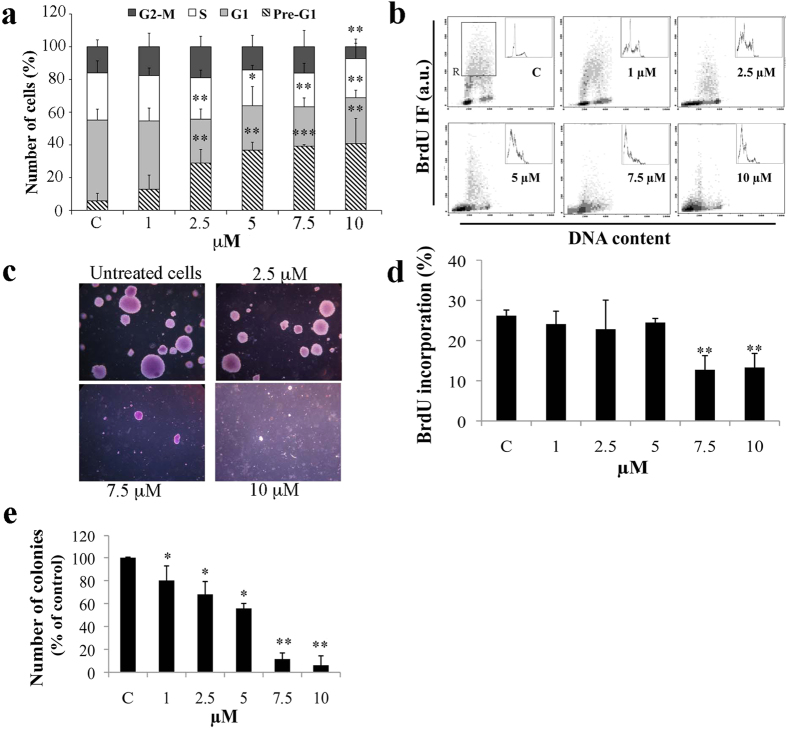
Effect of DHS on LLC cell proliferation. (**a**) Cell cycle phase distribution was evaluated by flow cytometry after staining with propidium iodide. (**b,d**) DNA synthesis was determined by a biparametric flow cytometry analysis of BrdU incorporation versus DNA content. Representative profiles of cell cycle distribution (**b**) and the relative quantification (**d**) of the percentage of BrdU incorporation calculated in the S phase region (R). (**c,e**) Anchorage-independent growth was determined by a soft agar assay. Treatments were made directly by adding DHS to cell suspension together with the semi-solid agar. Representative images of DHS-treated LLC colonies in soft agar (**c**) were taken after 21 days of growth by phase contrast microscopy using ×20 magnification objective. The number of colonies in soft agar was counted and relative quantification was performed (**e**). Data are the means ± SD from at least three independent experiments: values are expressed as percentages and are relative to untreated control cells (*p ≤ 0.05 and **p ≤ 0.01 compared with control cells).

To investigate the effect of DHS on cell proliferation, the distribution of LLC cells in each phase of the cell cycle was analyzed by flow cytometry (Fig. 2a). DHS-treated cells significantly accumulated in the pre-G1 hypodiploid region of the DNA profile, which correspond to the level of apoptotic cells. This accumulation occurred in a dose-dependent manner, achieving 13% at 1 μM until 41% at 10 μM of DHS. Concomitantly, a significant reduction in G1-phase cells was detected at all DHS concentrations, except for the highest one, in which also G2+M-phase cells were reduced by about 50%. To further investigate whether the cell cycle imbalance induced by DHS was accompanied by DNA synthesis inhibition, DNA replication was assessed by bivariate flow cytometry DNA/BrdU analysis (Fig. 2b). A significant reduction in BrdU incorporation by about 50% (p ≤ 0.01) at both 7.5 and 10 μM was detected, compared to untreated control cells, thereby indicating a strong inhibition of DNA synthesis by DHS (Fig. 2d). A dose-dependent peak at the pre-G1 region was also confirmed by this assay.

**Figure 3:**
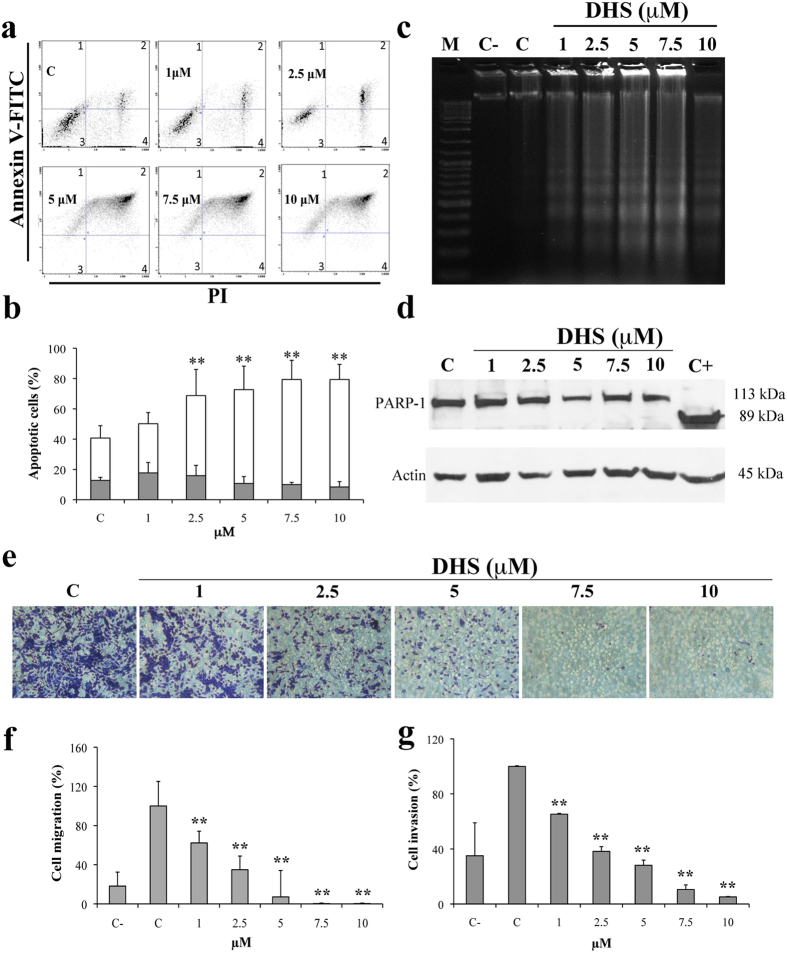
DHS and apoptosis. (**a**) Representative cytograms of Annexin V-FITC versus PI fluorescence intensities, as determined by flow cytometric analysis in LLC cells at different concentrations of DHS. Within each histogram, the quadrants 1 and 2 represent early and late apoptotic cells, respectively, the quadrant 3, viable cells and the quadrant 4, necrotic cells. (**b**) Quantification of early and late apoptotic cells. Data are the means ± SD from at least three independent experiments: values are expressed as percentages and are relative to untreated control cells (*p ≤ 0.05 and **p ≤ 0.01 compared with control cells). (**c**) Internucleosomal DNA degradation in LLC treated cells. (**d**) Western blot analysis of apoptotic marker (PARP-1 cleavage). LLC cells treated for 24 h with increasing concentrations of DHS and HeLa cells deprived of serum and used as positive control (C+) are shown. Actin as loading control is also reported. (**e**) Representative images of stained LLC cells after migration in the presence of increasing concentrations of DHS. (**f**,**g**) LLC migration and invasion data obtained from three independent experiments, as evaluated through the use of the Boyden chamber and filters treated with collagen type I and matrigel, respectively; values are expressed as percentages and are relative to untreated control cells. (*p ≤ 0.05 and **p ≤ 0.01 compared with control cells).

To assess whether cell cycle impairment influences anchorage independent growth of LLC cells, we performed cell clonogenic efficiency in agar. Cell treatments with DHS induced a 20, 32 and 45% mean reduction in the number of colonies, at 1, 2.5 and 5 μM, respectively; whereas, a total inhibition was observed at 10 μM (Fig. 2c,e). All these results indicate that DHS can strongly affect LLC cell proliferation, both by inhibiting DNA synthesis and driving cells towards apoptotic pathway. To confirm apoptosis, we next performed different approaches. Morphological features, such as condensation of chromatin and nuclear fragmentations, were observed at 5, 7.5 and 10 μM of DHS by using Hoechst 33258 staining (data not shown). Then, flow cytometric annexin V-FITC and PI staining (Fig. 3a,b) evidenced a significant increase in the late apoptosis, starting from 2.5 μM of DHS, in which 53% of the cells were apoptotic (p ≤ 0.01), compared to control cells (28%). At the biochemical level, DNA fragmentation was also detected (Fig. 3c), showing a direct correlation with flow cytometric data. Unexpectedly, PARP-1 proteolysis analysis by Western blot did not evidence PARP-1 cleavage in all DHS-treated cells (Fig. 3d).

### DHS inhibits migration and invasion of LLC cells

In the Boyden chamber assay, the treatment with 1, 2.5, 5, 7.5 and 10 μM of DHS strongly inhibited the migration of LLC cells by approximately 40, 65, 92% at the lower concentrations, achieving a 100% inhibition at 7.5 and 10 μM (Fig. 3e,f). Figure 3g shows the similarity between the invasiveness of DHS-treated cells and their migratory ability. In fact, the invasiveness of LLC cells was significantly reduced in the presence of DHS, at all concentrations used, and almost completely at 10 μM.

### DHS reduces primary tumour size and angiogenesis in a mouse model

**Figure 4:**
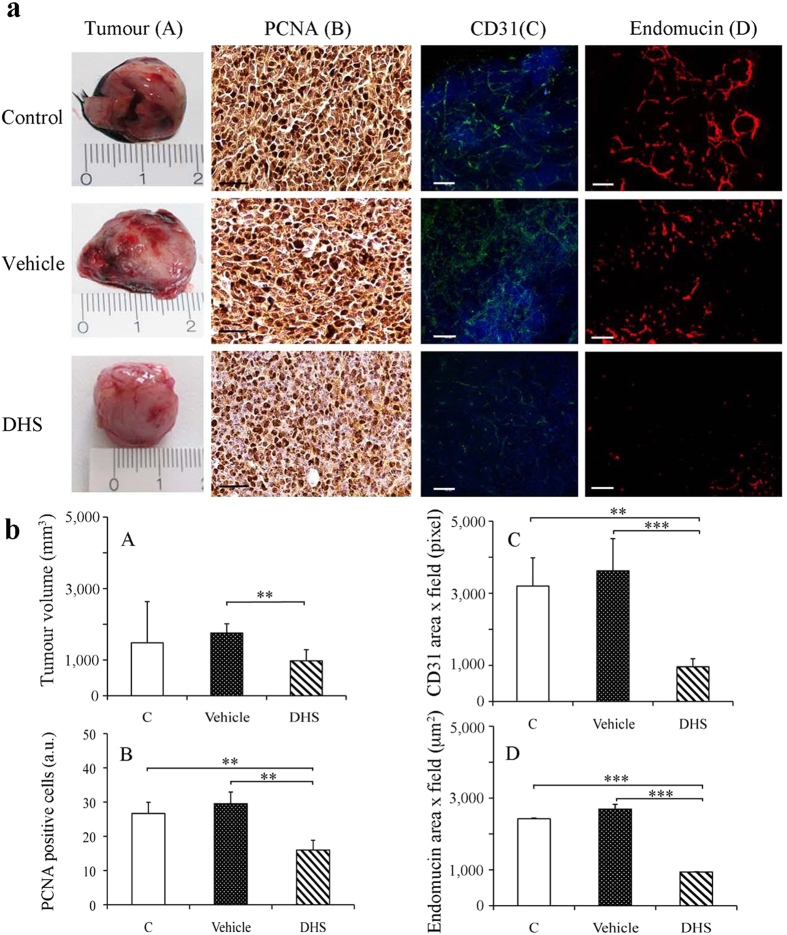
Tumour size and angiogenesis in a mouse model after DHS treatment. (**a**A) Macroscopic representative LLC primary tumours in control and 4 weeks DHS- and ethanol-treated mice and the corresponding tumour growth rates (**b**A). (**a**B) PCNA representative images obtained after immunostaining of primary tumour masses with PCNA antibody and DAB detection (bar = 50 μm) in control, vehicle- and DHS-treated mice and (**b**B) the relative quantification of PCNA-stained positive cells. (**a**C) Representative images of CD31 whole mount staining (bar = 100 μm) and quantification as obtained by confocal microscopy (**b**C). (**a**D) Endomucin immunofluorescence staining of primary tumour masses (bar = 100 μm) and relative quantitative analysis (**b**D). 15–18 mice/group were used; data shown are means ± SEM of 5 independent experiments (n = 5). (*p ≤ 0.05, **p ≤ 0.01 and ***p ≤ 0.001).

Prior to inoculation of C57BL/6J mice with a single-cell injection of LLC cells, we administered DHS (25 mg/Kg/day) for 7 days in drinking water. The administration to LLC bearing-mice continued for 21 days during which neither acute toxicity nor side effects, such as lethargy and sickness, of treated-mice was ascertained. In addition, no significant difference of the body weight between vehicle- and DHS-treated group was detected: 24.7, 23.6 and 23.6 g were the final mean values for control, vehicle and DHS groups, respectively. DHS-drinking mouse survival at the end of the 28-day study was 95%; the control mice survival for the same period was 85%. Local tumour growth was monitored every day for 3 weeks, and the primary masses were explanted and measured by a calliper at the end of treatments. The mean of the tumour volumes in DHS-treated group was significantly decreased, by 37% compared to the vehicle group (p ≤ 0.01), and by 10% with respect to the control group (Fig. 4aA,bA). To assess the potential effect of DHS against *in vivo* tumour growth, paraffin-embedded primary masses were sliced and sections were immunostained for PCNA, an endogenous cell proliferation marker^30^. As shown in Fig. 4aB,bB, PCNA-stained positive cells in DHS-treated group were significantly decreased by 50% with respect to both control and vehicle groups (p ≤ 0.01).

Since angiogenesis is absolutely required to promote tumour growth, invasion and metastasis^31^, *in vivo* evidence for anti-angiogenic effects of DHS treatment was investigated by immunostaining of the tumour sections for two endothelial cell markers, such as PECAM-1, known as CD31, and endomucin (Fig. 4aC,D). Both these proteins are highly expressed when endothelial cells exhibit angiogenic phenotype. Using the whole mount staining on slides of fresh tumour tissue, through the construction in 3-D with the confocal microscopy, the presence and integrity of the blood vessels was considered. Tumour vascular density detected by CD31 staining was significantly reduced of about 70% in DHS-treated group (Fig. 4bC). Similarly, numerous endomucin-positive cells were observed both in control and vehicle-treated tumours, whereas in DHS treated mice, few red spots were detectable in the tumour masses (Fig. 4aD). The number of microvessels in DHS-treated tumours was reduced by 2.5 fold with respect to the control and vehicle groups (Fig. 4bD). Collectively, these results demonstrated that DHS markedly inhibits tumour angiogenesis *in vivo*.

### DHS inhibits tumour dissemination both in mouse and in *zebrafish* models

**Figure 5:**
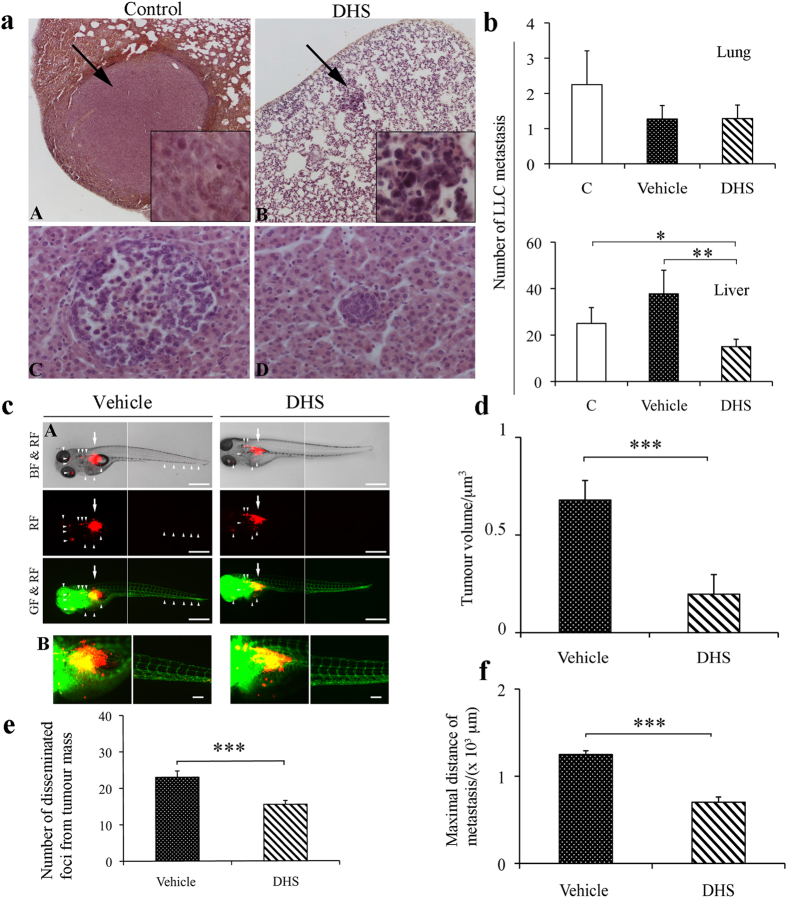
LLC cell dissemination both in mouse and in *Tg(fli1:EGFP)* zebrafish tumour models. (**a**,**b**) Tumour cell dissemination and metastases detected after 4 weeks of mice treatments. Representative image of lung and liver metastases are shown in (panel A,B and C,D), respectively. Arrows indicate metastases in lung. (**b**) Quantification of number of LLC metastases in lung and liver (15–18 mice/group). The data shown are means ± SEM of 5 independent experiments (n = 5). (*p ≤ 0.05, **p ≤ 0.01). (**c**) Panel A, LLC cells were implanted into 48 h post-fertilization zebrafish embryos. Tumour cell dissemination and metastases were detected at day 4 after injection. Arrows indicate primary tumours, white arrowheads indicate disseminated tumour foci (bar = 500 μm). BF: bright field; GF: green fluorescence; RF: red fluorescence. B, representative 3-D micrographs of confocal images of tumours (red) and vasculature (green). Yellow signal show the vasculature overlapping with tumour cells (Scale bar, 100 μm). (**d**) Quantification of tumour volume (n = 20/group). (**e**) Quantification of number of disseminated tumour foci (n = 20/group). (**f**) Average of maximal distances of metastatic foci (n = 20/group). Data shown are means ± SEM of 3 independent experiments (n = 3). (*p ≤ 0.05, **p ≤ 0.01 and ***p ≤ 0.001).

**Figure 6:**
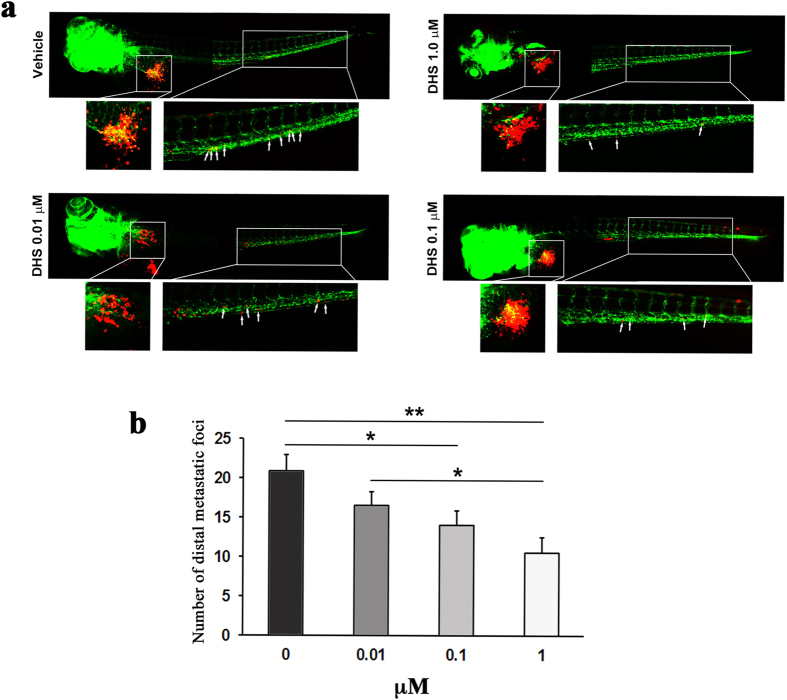
DHS inhibits metastasis of LLC cells in a dose-dependent manner. (**a**) Fluorescent micrographs of DiI-labeled LLC cells (red) and host endothelial cells (green) in 5 days old fli1:EGFP transgenic zebrafish embryos, 3 days following tumour cell implantation and treatment with vehicle, DHS at 0.01 μM, DHS at 0.1 μM or DHS at 1 μM. The tumour and main metastatic site in the caudal venous plexus are indicated by white boxes and shown in higher magnification below. White arrows indicate metastasized tumour cells. (**b**) Quantification of the number of metastatic foci in the caudal venous plexus, 3 days after LLC implantation. *p ≤ 0.05, **p ≤ 0.01. n = 20, 15, 22 and 15 for vehicle-treated, DHS 1 μM, DHS 0.1 μM and DHS 0.01 μM groups respectively.

To investigate the activity of DHS on cancer cell motility and metastasis formation, both LLC murine tumour and zebrafish embryos models were used. Figure 5a shows representative images of metastasis to lung (A, B) and liver (C, D), obtained after haematoxylin and eosin staining. DHS treatment determined a significant 40% mean reduction in the number of liver metastasis compared to the control group (p ≤ 0.05), and 60.2% compared to the vehicle-treated mice (p ≤ 0.01, Fig. 5b). Conversely, the number of tumour cell colonies metastasizing to the lung was very low (1 to 3/group) and they did not achieve a statistically significant variation. Nevertheless, when lung metastases were present, their dimensions were bigger, as shown in the representative image in Fig. 5aA, than those in the liver (Fig. 5aC). Interestingly, in DHS-treated mice, metastases appear to be much smaller than those of control and vehicle-treated mice (Fig. 5aB,D), both in the liver and lung. In addition, to investigate possible mechanisms of liver metastases inhibition, epithelial-mesenchymal transition was evaluated by using EMT markers, such as E-cadherin and vimentin, but no difference between control and DHS-treated animal metastases was highlighted (Supplemental Fig. S1). The activity of DHS on metastatic behaviour of LLC cells was also investigated in zebrafish embryos after injection into perivitelline cavity of LLC cells labelled *in vitro* with DiI dye. As shown in Fig. 5c,d, in tumour-bearing fish embryos, the size of primary tumour of DHS group was significantly reduced by the treatment with respect to the vehicle one (by about 72%, p ≤ 0.001). In addition, a substantial number of tumour cells in vehicle group zebrafish embryos were significantly disseminated away from primary sites towards distal parts of the fish body, including the head and tail regions, reaching the maximal distance of metastasis in comparison with DHS treated group (Fig. 5c,f). High-resolution image analysis allowed detecting single tumour cells in distal part of the fish body (Fig. 5c). Quantification analysis showed that the number of disseminated foci from tumour mass was reduced (31%) by the molecule with respect to the vehicle group (Fig. 5e). Looking into the dose-dependent effects of DHS we found that while a concentration of 0.01 μM DHS did not significantly inhibit distal metastasis of LLC cells in zebrafish embryos, treatment with 0.1 μM DHS significantly inhibited metastasis (32%) albeit slightly less than after treatment with 1 μM DHS (49%), compared to vehicle (Fig. 6). 10 μM DHS were toxic to the zebrafish embryos, indicating that the best effect is observed at the maximally tolerated dose of 1 μM.

### Plasma HPLC/UV/MS detection of DHS

**Figure 7:**
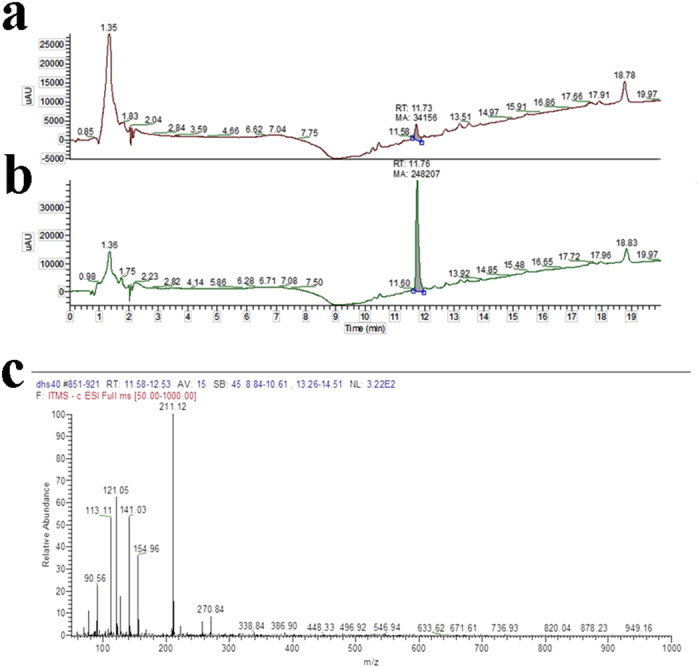
(**a**) Representative UV-HPLC chromatogram of the plasma sample of mice treated with DHS (25 mg/Kg/day) for 28 days. (**b**) UV-HPLC chromatogram of the plasma sample of mice treated with DHS overlaps to the standard (final concentration 10 ng/mL). (**c**) MS/MS spectrum of DHS, ion m/z 211, corresponding to [M − H]^−^. Three independent experiments have been performed.

DHS was detectable in mice plasma at the end of the treatment at the concentration of 5 ng/mL as evidenced in Fig. 7a. The identity of the peak at the retention time of 11.73 min comparable with the retention time of the standard subsequently injected was shown (Fig. 7b,c).

## Discussion

In agreement with our previous evidence^9^,^13^, the study presented here has confirmed DHS as an effective agent in suppressing both anchorage-dependent and -independent proliferation of LLC cells. This inhibition appears to be consistent with DHS concentration and, in part, ascribed to decreased DNA synthesis given that a significant reduction in BrdU incorporation was found in DHS-treated LLC cells. DNA synthesis reduction, in turn, may be related to pol δ inhibition by DHS, as we have already demonstrated in *in vitro* assays^9^. Moreover, a loss of cell viability was detected in LLC cells, even at the concentration as low as 2.5 μM, with occurs through apoptosis as detected by DNA fragmentation analysis and annexin V staining. LLC cells die in a dose-dependent manner with a mechanism independent by caspase 3, as well as caspase 8 and 9 (data not shown). In our previous study on normal and cancer human cells no apoptosis was observed in the presence of DHS^9^,^13^, and no data are available in the literature to state a different effect of this compound on cells deriving from different species. However, in agreement with our results, similar increasing in apoptotic death was reported in resveratrol-treated LLC cells by Kimura *et al*.^32^.

The efficacy of DHS on LLC cells *in vitro* prompted us for *in vivo* application. In LCC-bearing C57BL/6J mice we found that tumour growth and metastases formation were inhibited by DHS administered in their drinking water (25 mg/kg/day). Nevertheless, the plasma DHS levels, as determined by HPLC/UV/MS at the end of mice treatment, were 5 ng/mL, lower than the expected one. Very recently, pharmacokinetic studies have shown a maximum concentration of 15.3 or 356 ng/mL in rat plasma, after a single oral administration of DHS suspension in 0.3% sodium salt of carboxymethylcellulose or a solution prepared with 2-Hydroxypropyl-β-cyclodextrin, respectively, both at the dose of 10 mg/Kg^33^. However, the two model systems are not comparable, since they differ in animals used (rat vs. mouse), beyond of oral single administration against daily consumption of DHS in ethanol/water solution (1/99, VV^−1^). We have also to take into account that, based on the total volume drunk by DHS-treated mice in 28 days of administration, each animal took in about 2.5 mg of the compound, corresponding to the 0.35% of the dose chosen in our model, thus explaining the low levels of DHS found in the plasma. Muzzio *et al*.^34^ reported much more comparable plasma concentrations of resveratrol after a long-term treatment of dogs. In fact, 13 weeks of oral administration with 200 mg/kg/day determined a plasma concentration ranging between 1.7–2.6 μg/mL. A large number of studies on resveratrol bioavailability, both in animals and humans, have been conducted as reviewed^3^,^35^,^36^; it should be noted that resveratrol concentrations in tissues and organs depends on the route of administration, duration of treatment, and animal species. However, one documented problem is its limited bioavailability owing to its rapid metabolism in the liver towards derived sulfate and glucoronide metabolites^37^,^38^. In fact, after a single oral dose treatment of 25 mg in human volunteers, only small amounts of free resveratrol (≤5 ng/mL) were detected, whereas high level of metabolites (400–500 ng/mL) were found^37^. Recently, it has been reported that methylated polyphenols possess an increased *in vivo* stability^39^. In fact, the dimethyl ether analogue of resveratrol, pterostilbene, that shows similar antioxidant, antiproliferative and antitumour activity^6^,^40^,41, has a bioavailability of 80% in rats vs. 20% for resveratrol^42^. Similarly, the only pharmacokinetic studies of resveratrol and DHS assessed in the same animal model, with similar formulation, indicated that DHS possess a better pharmacokinetic profile^33^,^43^ than the parental molecule. However, we found two metabolites of DHS that are probably attributable to the glucuronide-sulfate and disulfite of DHS (data not shown). Despite its low concentration in plasma, the tumour volume of DHS-drinking animals was significantly lower than that of the vehicle-treated group, in agreement with data obtained in the same experimental model on mice treated with resveratrol^32^,^44^. The PCNA-labelling index was significantly reduced, as already reported by Kimura *et al*. in colon 26-bearing mice^26^, thus confirming the antiproliferative properties of DHS in our *in vivo* model. Furthermore, a marked anti-angiogenic effect was observed on primary masses, where a reduction in both CD31 and endomucin (neovascularisation markers)-positive area was clearly observed. These findings indicate that the antitumour action of DHS may be also related to an angiogenesis inhibition, in agreement with the observed inhibition of HUVEC cell proliferation in *in vitro* experiments (Supplemental Fig. S2). Comparable results were obtained in the same animal model by using resveratrol^32^,^45^, and in colon 26-bearing mice by DHS^26^. Metastatic capacity is a fundamental characteristic of malignancy, it is subject to genetic regulation, distinct from that of tumorigenesis, and it is crucial to the survival of the host. It has been demonstrated that DHS can contrast the *in vitro* migration and invasion of human breast cancer cells^13^. In the present study, through the use of the Boyden chamber, it was possible to assess the ability of DHS to interfere with migration and extracellular matrix overstepping of LLC cells, a process carried out actively by tumour cells that allows them to infiltrate tissues. These *in vitro* data appear to match our *in vivo* results, in which DHS negatively affects metastasis dissemination in the liver; instead, no involvement of epithelial-mesenchymal transition was observed. Finally, the trend towards the reduction observed in lung metastases could be dependent on the insufficient concentrations or non-reactive forms of DHS in the lungs of mice. The results obtained in the zebrafish model highlight the limitation of the low plasma concentration in mice model. In fact, DHS significantly reduced the size of the primary tumours derived from LLC cells injected as well as the number of disseminated foci to the distal parts of the fish body in a dose-dependent manner with the maximal effects being observed at 1 μM, without over toxicity. In this model, however, significant effects were observed already at 0.1 μM corresponding to a plasma concentration of 21.2 ng/mL, which might be achievable in *in vivo* experiments. Overall, the data indicate that DHS holds great promise in the field of chemoprevention by natural agents, and further preclinical studies are needed to improve its delivery to tumour masses or specific sites of the body, at specific times, allowing to reach the effective concentration, as established in *in vitro* studies.

## Methods

### Reagents, cell cultures and treatments

4,4′-dihydroxy-*trans*-stilbene (DHS) was synthesized as described^46^. Murine LLC cells, provided from Zooprofilattico Institute of Brescia, were cultured in D-MEM supplemented with 8% FBS, 200 mM L-glutamine, 100 IU/mL penicillin and 100 μg/mL streptomycin, all obtained from Gibco Invitrogen. HUVEC cells, kindly provided by Prof. J. Majer, were cultured as previously reported^47^. LLC or HUVEC cells were treated for 24 h with 1, 2.5, 5, 7.5 and 10 μM of DHS. One hundred mM stock solution of this compound was prepared in DMSO and diluted directly in cell culture medium. Final concentration of DMSO did not exceed 0.15% (v/v) and control cells were treated with the same concentration of vehicle that did not exert any effect in all the assays.

### Cytotoxicity, cell cycle and apoptosis analysis

The cytotoxicity of DHS was evaluated by MTT assay and Trypan Blue staining, as previously described^13^. Cell cycle experiments were performed by using 5-bromo-2′-deoxyuridine incorporation (BrdU), as previously reported^9^. Annexin V/FITC staining was obtained by processing LLC cells as indicated in the protocol provided by the supplier (eBioscience), then analysed by flow cytometer Coulter Epics XL (Coulter Corporation, USA). DNA isolation and analysis by agarose gel electrophoresis were performed as described^48^. For PARP-1 proteolysis, LLC cells were treated with DHS, and each sample was harvested and processed by Western blot, as previously published^49^. Transferred membranes were probes with anti-PARP-1 polyclonal antibody (215–228) (Calbiochem), followed by anti-rabbit HRP (Calbiochem). Densitometric analysis was conducted using the public domain NIH-Image program available on Internet at http://rsb.info.nih.gov/nih-image.

### Anchorage-independent growth, cell migration and invasion

LLC cells (2 × 10^4^) in 500 μl of D-MEM (20% FBS) were mixed to 500 μl of 0.33% Bacto Agar (Difco Laboratories, Detroit, MI) containing increasing concentrations of DHS. Each mixture was poured in culture cell dishes previously prepared with 5 mL of 0.6% Bacto agar in complete D-MEM and incubated at 37 °C for 2 weeks. Five hundred μl of D-MEM with 60% FBS were added twice a week to each cell dish. The colonies formed were stained with 500 μl of 0.005% Gentian Violet for 1 h, counted using a 10X magnitude inverted microscope (Leitz DM-IL, Leica). To determine LLC cell migration or invasion, the Boyden chamber (Neuroprobe, Gaithersburg, MD) was assembled by inserting collagen - (100 μg/mL) or Matrigel (200 μg/mL, BD Biosciences)-coated filters, respectively, as previously described^13^.

### Murine tumour model and treatments

Sixty male C57BL/6J mice (4 weeks old) were purchased from Harlan Laboratories (Udine, Italy) and, according to the ethical guidelines of the University of Pavia, were housed (*Centro Interdipartimentale di Servizio per la Gestione Unificata delle Attività di Stabulazione e di Radiobiologia*) and maintained under standard conditions of a 12 h dark/12 h light cycle, a temperature of 24 ± 2 °C, and relative humidity of 50 ± 10%. DHS (25 mg/kg/day) was added to drinking water (1% Et-OH/water), replaced with fresh DHS solution three times a week. Water volumes were constantly checked for the duration of the experiment. All experimental procedures were in accordance with the European Convention for Care and Use of Laboratory Animals and were approved by the local Animal Ethic Committee of the University of Pavia (Document n. 1, 2012). Mice were divided into 3 groups: positive control (LLC tumour-bearing mice), vehicle (mice taking 1% ethanol), and mice drinking DHS. Treatments started a week before injecting tumour cells, and continued until the sacrifice day. A single-cell suspension (1 × 10^6^), in 400 μl of saline buffer, was implanted subcutaneously in the left side of each animal, after locally anaesthetizing. About three weeks later the injection, the animals were killed by a lethal dose of ether, then tumour masses, livers and lungs were collected, dissected and fixed with 4% formaldehyde in phosphate buffered saline (PFA) (Carlo Erba) for histological analysis. Primary tumours were measured by a calliper, and their volume calculated according to a standard formula (length × width^2^ × 0.52)^45^. The number of metastases was counted both in livers and lungs. From each mouse, about 300 μl of plasma were separated by centrifugation after the sacrifice for biochemical analysis.

### Hystology and immunohistochemistry

Fresh tissues were fixed with 4% (wt/vol) PFA overnight, paraffin-embedded or processed for whole-mount immunohistochemical analysis. Some tissue samples were stained with H&E using a standard protocol (haematoxylin and eosin G, Sigma Aldrich and Merck Certistain, respectively). Number of lung and liver metastases was counted by scoring slides under an optic microscope (Leitz), and photographed under a digital microscope Nikon Eclipse 80i with a camera Nikon Digital Sight DS-Fi1. Epithelial-mesenchymal transition was evaluated in LLC liver metastases by antibodies against E-cadherin and vimentin, as previously reported^50^. PCNA (PC10, Dako) staining was performed using M.O.M.^TM^ reagent kit. The sections were counterstained with haematoxylin for 15 min, embedded with Eukitt (O. Kindler GmbH), and images analysis was performed using ImageJ software. For endomucin staining, the paraffin-embedded sections were stained at 4 °C o.n. with the specific rat anti-mouse antibody, followed by Alexa 555 goat anti-rat secondary antibody (both 1:400). Samples were then mounted in Vectashield mounting medium (Vector Laboratories, Inc.), and stored at −20 °C. Confocal microscope (Nikon Eclipse C1) images of 6–10 randomized fields were collected and analysed using Adobe Photoshop CS4 software. Both endomucin immunostaining and zebrafish experiments (see below) were carried out at the Karolinska Institute (Stockholm). For whole mount staining, fresh tumour tissues were harvested, fixed in 4% PFA and processed as described^51^.

### Biochemical analysis

To quantify DHS in the plasma of mice, the method for resveratrol detection by Muzzio *et al*.^34^ was adapted and performed for its analogue. HPLC-UV-ESI/MS and analyses have been carried out on a ThermoFisher Scientific HPLC/UV/MS system (Thermo Scientific LCQ *FLEET*). Separation of DHS from plasma components was achieved using a Luna C18 3μm 2×100 mm column maintained at r.t., with a flow rate of 0.2 mL/min, and injection volume 20 μl. The mobile phase consisted of 5 mM ammonium acetate in water containing 2% propan-2-ol and methanol with 2% propan-2-ol. A mobile phase gradient was performed. An Electro Spray Ionization (ESI) interface was used as ion source, operating both in negative and in positive ion mode. Acquisition was performed in full scan mode (mass range 50–1000 Da). Ion spray voltage, capillary voltage were −5000V and −0.5 V in negative ion mode and + 5000V and +35V in positive ion mode. The capillary temperature was 220 °C. The DHS quantification in plasma was performed using a calibration standard curve (5–5000 ng/mL) starting from stock solution (100 mM) in DMSO.

### Zebrafish tumour model

The experiments on zebrafish model, were performed in the laboratory of Prof. Y. Cao at the Karolinska Institute of Stockholm, as described by Yang *et al*.^52^. All experimental procedures of zebrafish research were approved by the Northern Stockholm Experimental Animal Ethical Committee. Methods were carried out in accordance with the approved guidelines. Zebrafish transgenic strain expressing enhanced green fluorescence protein (*EGFP*) under the fli1 promoter (*fli1:EGFP*) were used as a tumour model. For cell injection, zebrafish embryos anesthetized with 0.04 mg/mL of tricaine (MS-222, Sigma) were transferred onto a modified agarose gel for microinjection with LLC cells. Approximately 100 DiI (Invitrogen; catalog no. D3899)-labelled cells were injected into perivitelline space of each embryo using an Eppendorf microinjector (FemtoJet 5247, Eppendorf and Manipulator MM33-Right, Märzhäuser Wetziar). Non-filamentous borosilicate glass capillaries needles were used for the microinjection (1.0 mm in diameter, World Precision Instruments, Inc.). Then, fish embryos were immediately transferred into PTU water, checked one by one and pictures were taken using a fluorescent microscope (Nikon Eclipse C1). Only the zebrafish embryos with a single localizated injection into perivitelline space were chosen and each zebrafish embryo was put in a well of a 48-well plate with 500 μl of PTU at 28 °C, in order to test DHS at the indicated concentrations dissolved in 0.5% ethanol. For imaging and analysis, Zebrafish embryos were carefully placed onto the gel cushion, in a small drop of 0.04% tricaine. Tumour growth and invasion were examined at days 0 and 4 using fluorescent microscope. Disseminated tumour cells per embryo were quantified in 10 zebrafish embryos per group.

### Statistical analysis

Experimental data were presented as mean determinants ± SEM or SD and analyzed using a two-tailed Student t-test. Statistical P values were presented as follows: *p ≤ 0.05, **p ≤ 0.01 and ***p ≤ 0.001 were considered to be statistically significant.

## Additional Information

**How to cite this article**: Savio, M. *et al*. Resveratrol analogue 4,4'-dihydroxy-*trans*-stilbene potently inhibits cancer invasion and metastasis. *Sci. Rep*. **6**, 19973; doi: 10.1038/srep19973 (2016).

## Supplementary Material

Supplementary Information
