# Supplementary material for: Resveratrol analogue 4,4′-dihydroxy-trans-stilbene potently inhibits cancer invasion and metastasis
Source: Sci Rep. 2016 Feb 1;6:19973. doi: 10.1038/srep19973 (PMC4734289; doi:10.1038/srep19973)
Supplement: Supplementary Information [file srep19973-s1.pdf]

Resveratrol analogue 4,4'-dihydroxy-*trans*-stilbene potently inhibits cancer invasion and metastasis

Monica Savio[+1]*, Daniela Ferraro[+1], Cristina Maccario[1], Rita Vaccarone[3], Lasse D. Jensen[2,4], Federica Corana[5], Barbara Mannucci[5], Livia Bianchi[1], Yihai Cao[2] and Lucia Anna Stivala[1]*

[1]Department of Molecular Medicine, Immunology and General Pathology Unit, University of Pavia, Pavia, Italy;

[2]Department of Microbiology, Tumour and Cell Biology, Karolinska Institute, Stockholm, Sweden;

[3]Department of Biology and Biotechnology, Comparative Anatomy and Citology Laboratory, University of Pavia, Pavia, Italy;

[4]Department of Medical and Health Sciences, Linköping University, Linköping, Sweden

[5] Centro Grandi Strumenti, University of Pavia, Pavia, Italy.

[+]These authors contribute equally to this work

*Corresponding authors: monica.savio@unipv.it, luciaanna.stivala@unipv.it

EMT transition

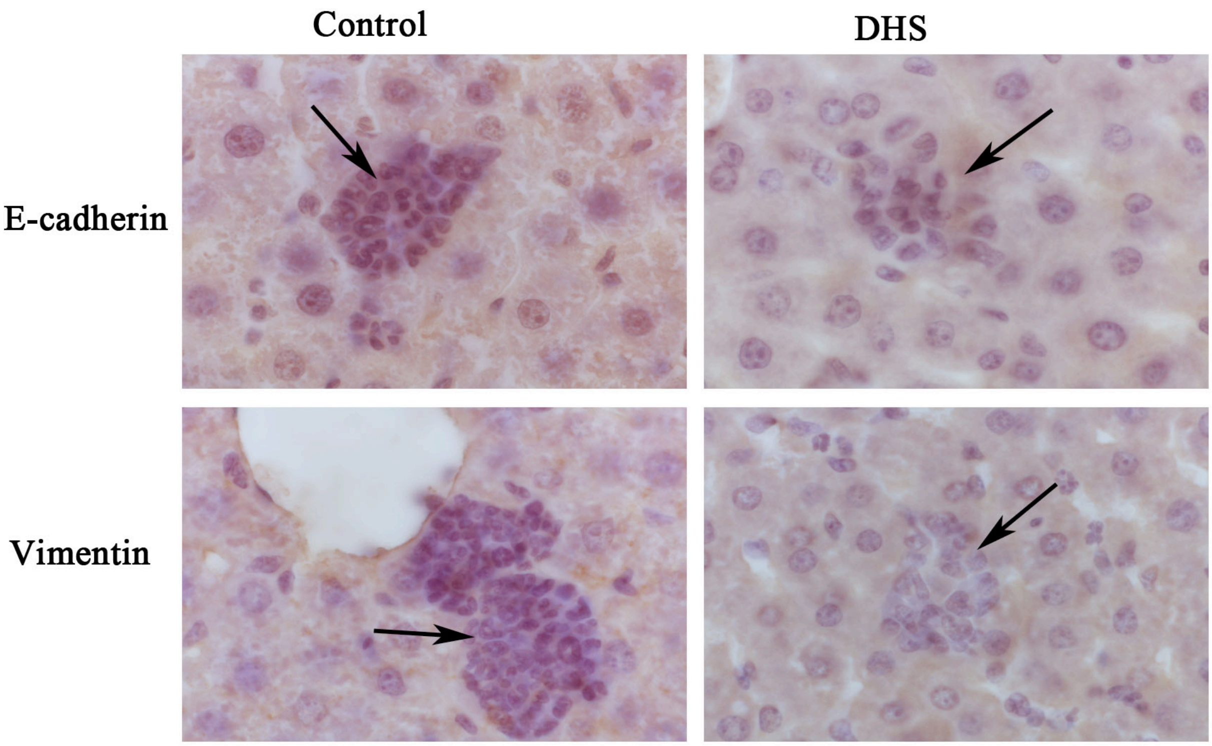

Epithelial-mesenchymal transition, evaluated by using E-cadherin and Vimentin antibodys, was not detected both in control and DHS-treated liver metastases (arrows, X100 magnification objective).

Supplemental Figure S1

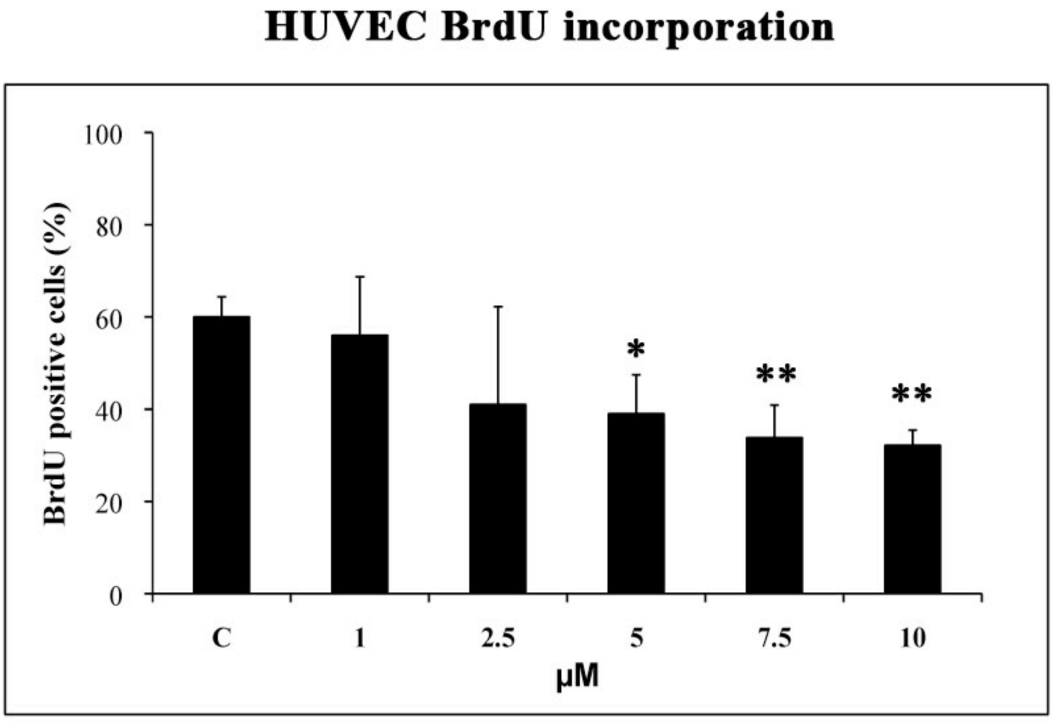

DHS significantly inhibited BrdU incorporation starting from 5 μM with a reduction of 35% with respect to HUVEC control cells ($p<0.05$). In the range 30-60-90 μM DHS inhibited completely (100%) the DNA synthesis (data not shown).

Supplemental Figure S2
